# Advancing Global Health Equity in the COVID-19 Response: Beyond Solidarity

**DOI:** 10.1007/s11673-020-10008-9

**Published:** 2020-08-25

**Authors:** Stephanie B. Johnson

**Affiliations:** 1 Wellcome Centre for Ethics and Humanities, University of Oxford, Oxford, UK; 2 Ethox Centre, University of Oxford, Oxford, UK

**Keywords:** Global health justice, COVID-19, Ethics

## Abstract

In the coming weeks and months SARS-CoV-2 may ravage countries with weak health systems and populations disproportionately affected by HIV, tuberculosis (TB), and other infectious diseases. Without safeguards and proper attention to global health equity and justice, the effects of this pandemic are likely to exacerbate existing health and socio-economic inequalities. This paper argues that achieving global health equity in the context of COVID-19 will require that notions of reciprocity and relational equity are introduced to the response.

While much of the world’s attention is currently focused on Europe and the United States, experts worry that in the coming weeks and months SARS-CoV-2 may ravage countries with weak health systems and populations disproportionately affected by HIV, tuberculosis (TB), and other infectious diseases (Nordling 2020).

At the same time, the Trump administration has been accused of offering a German medical company “large sums of money” for exclusive access to a coronavirus disease (COVID-19) vaccine (Oltermann 2020). According to an anonymous source, Trump was aiming to secure a vaccine against the coronavirus for the United States, “but for the US only.” It was also reported that the German government was offering its own financial incentives for the vaccine to stay in the country. This stark example reveals the unfairness and inequities of the global political and health systems. Unfortunately, inequitable distribution of vaccines is not the only way in which global inequities can be reproduced. Without safeguards and proper attention to global health equity and justice, the effects of this pandemic are likely to exacerbate existing health and socio-economic inequalities.

Global coordinated efforts in response to COVID-19 led by international organizations such as UNICEF and the World Health Organization (WHO) have attempted to integrate notions of global “solidarity” into practice and policy. In this paper we explore how solidarity is used in this context and how it can be manifest. We argue that achieving global health equity in the context of COVID-19 will also require that other equity-orientated perspectives, namely reciprocity and relational equity, must be introduced to the response.

## Solidarity

In April 2020, the UN General Assembly unanimously adopted a resolution calling for increased global solidarity and international cooperation against the novel coronavirus outbreak. Similarly, in April 2020 the WHO and UNICEF initiated the COVID-19 Solidarity Response Fund. UNICEF (2020) reports that “money collected through the fund will be used, among others, to train and equip communities and health-care workers to prevent, detect and treat COVID-19. It will help countries expand their health-care capacity and mitigate its social impact.” Similarly, the WHO publication *Addressing Human Rights as Key to the COVID-19 Response* sets out that

[u]nder international human rights law, the obligations undertaken by State parties beyond their borders, i.e. to International Assistance and Cooperation are akin to their domestic obligations, not subsidiary or secondary in any way. COVID-19 is a reminder, not only of the global connectedness of the pandemic, but also of its solutions. Providing LMICs with international assistance and cooperation, both fiscal and technical, is crucial not only to individual nations’ efforts to address this pandemic but also to global efforts. (World Health Organization 2020, 3)

While “solidarity” is presented as the conceptual umbrella under which these moral and practical commitments fall, several reasons are offered for the importance of global solidarity: 1) it is legally required, 2) it will help LMICs address the outbreak in their own countries, and 3) global cooperation will also be instrumental in avoiding a second wave of infection in high-income countries. In this regard, solidarity is used instrumentally to avert crisis in high-income countries and also as a moral basis for a commitment to countries elsewhere.

The bioethical literature offers differing views on conceptual understanding of solidarity and the practical work that “global solidarity” might be able to do. In the context of global health, solidarity is often invoked normatively in connection with providing assistance to poor countries (Prainsack and Buyx 2012). Some, however, including Gostin et al. (2010), reject the notion of solidarity as “aid” and endorse an understanding of mutual assistance between countries motivated by a sense of shared duty, as distinct from charity (Prainsack and Buyx 2012):

Framing global health funding as “aid” is fundamentally flawed because it presupposes an inherently unequal benefactor-dependent relationship. Rather, global collaboration requires a collective response to shared risks and fundamental rights, where all states have mutual responsibilities. Charitable giving usually means that the donor decides how much to give, for what and to whom. Consequently, aid is not predictable, scalable or sustainable. It undermines the host country‘s ownership of—and responsibility for—health programmes. (Gostin et al. 2010, ¶8)

Prainsack and Buyx offer that of authors writing about solidarity and global health, most agree that solidarity should help to materialize a better distribution of resources and more equal access to healthcare across the globe. They also argue that “in its most bare-bones form, solidarity signifies shared practices reflecting a collective commitment to carry ‘costs’ (financial, social, emotional, or otherwise) to assist others” (Prainsack and Buyx 2012, 346). Solidarity is understood as a practice and not as an inner sentiment or an abstract value, one that can be reflected across three tiers: the individual, the institutional, and the legal and contractual (Prainsack and Buyx 2012). Dawson and Jennings deny that “costs” are a necessary requirement for solidarity. They “hold solidarity to be a deep and enmeshed concept, a value that supports and structures the way we in fact do and ought to see other kinds of moral considerations” (Dawson and Jennings 2012, 73) and suggest that “the foundational aspect of solidarity can be captured by the fundamental idea of ‘standing up beside’” (74). This is taken to have several key elements: solidarity requires a public action; the purpose of the action is orientated towards improving or correcting past or present disadvantage or injustice; and what is important is that action does not derive out of expectation of benefit from the other but out of moral concern for that other (Dawson and Jennings 2012).

What all this demonstrates is that it is “impossible to give an uncontroversial definition of solidarity” (Dawson and Jennings 2012, 72). Solidarity is conceptually contested; the concept often poorly defined in practice and by most accounts does not imply direct obligations. Solidarity can therefore fail to manifest in a meaningful way when aiming to advance global health equity. Further, solidarity does not incorporate important ethical and moral dimensions of global equity. That is to say “solidarity,” by most accounts, fails to account for the contributions that LMICs make to global health security and what they may be owed in return.

## Acknowledging Shared Contributions and Reciprocity

Theories of justice from political philosophy most often establish obligations for parties from high-income countries owed to parties from low- and middle-income countries (Pratt and Loff 2014). Yet, in recent decades, global collaborative efforts have formed a key part of addressing emerging infectious disease threats. The Global Health Security Agenda (GHSA), for example, is a group of countries, international organizations, NGOs, and private sector companies that have come together to advance a world safe and secure from infectious disease threats (GHSA 2020). Thirty-one countries around the world are partnering to reach the goals of the GHSA, under which nations make new, concrete commitments to elevate global health security as a national leaders-level priority (GHSA 2020). In the research domain, important work to develop globally compatible surveillance systems has been ongoing (Gardy and Loman 2018). Indeed, ongoing surveillance is a core part of COVID-19 global strategy.

Recognition of the required collaborative nature of disease detection means research practices have evolved within the scientific community, emphasizing openness, transparency, networks, and free exchange (Laird et al. 2020). The COVID-19 pandemic has seen unprecedented levels of collaboration and openness. NextStrain, for example, is an open repository that pulls in data from labs around the world that are sequencing SARS-CoV-2’s genome and centralizes it in a phylogenetic tree (Berditchevskaia and Peach 2020). Researchers have also been sharing new findings about the virus’s genomic profile through open source publications and preprint sites such as BioRxiv and Chinaxiv (Berditchevskaia and Peach 2020).

What all this demonstrates is that LMICs are not merely the recipients of knowledge and resources. As others have noted “northern researchers are not self-sufficient benefactors providing capacity-building and strengthening resources. They need the data, samples, skills, experience and expertise contributed by their Southern partners” (Parker and Kingori 2016). “Reciprocity is generally understood to be based on the notion of mutual regard and fair play. Reciprocity demands an appropriate balancing of the benefits and burdens of the social cooperation necessary to obtain the good of public health” (Viens et al., 2009, 211). Global pathogen threat detection systems create both burdens and benefits. Benefits may include creation of technologies and vaccines and increased healthcare and surveillance capacity in LMICs. Similarly, burdens may include use of resources, risks to privacy (through data-donation), and undesirable political or trade consequences. Countries that have participated in global surveillance and have borne the costs and burdens are owed something in return. This is true whether the downstream benefits of that participation have yet been realized or can be measured.

## Relational Equity

So far, we have taken a distributive view of justice. Relational egalitarians have proposed a different way of conceptualizing equity which focuses not on distributions as inherently important but instead on the quality of social relations among citizens and/or the ways in which social institutions “treat” citizens (Voigt and Wester 2015). In this context, other ways in which equity might be reflected in current and future pandemic responses is in the processes through which policies are designed and decided upon. Participation in decisions about public health policy in particular should increase the involvement of marginalized groups as “agents” of policy rather than merely recipients (Weinstock 2011). This could play out on many levels; here we focus on geopolitical issues and international science. Progress is required in this regard so that existing and historical injustices are not perpetuated or exacerbated.

Recent polling of the International Advisory Board of *The Lancet Global Health* on questions of authorship and recognition illustrate some of the challenges that continue to arise in ensuring fair involvement and recognition of researchers in LMICs. The greatest challenge being the greater power and resources of their partners and a lack of consensus on what is “fair” (*The Lancet Global Health* 2018). In January 2020, the Nuffield Council of Bioethics report *Research in Global Health Emergencies: Ethical Issues* (Nuffield Council of Bioethics 2020) highlighted the *Research Fairness Initiative Guide to High-Quality Reporting on Measures and Conditions that Promote Fair Research Partnerships* (Research Fairness Initiative 2018). Recommendations include, amongst others, early engagement of all partners; data ownership, storage, access, and use during and after research for LMIC partners; and specific measures to share intellectual property rights in collaborative research (Research Fairness Initiative 2018).

Of the more than three hundred clinical trials that have been launched to find a treatment for COVID-19, most are in China and South Korea, with more developing in the United States and Europe (Roussi and Maxmen 2020). Very few are taking place in Africa, Latin America, and South and Southeast Asia (Roussi and Maxmen 2020). Early trial entrants for the WHO’s Solidarity Clinical Trial, which seeks to compare the effectiveness of four drugs and drug combinations in treating COVID-19, are from Europe, where countries have some of the highest COVID-19 cases. But so far the only country in Africa to have formally joined the trial is South Africa (Roussi and Maxmen 2020). This has significant implications for the applicability of trial results to different populations.

If the benefits of research are to be achieved and a just global response to COVID-19 realized, this will be dependent upon both distributive and relational concerns. A successful global research effort will require attention to the ways in which all partners are treated and the ways in which priorities, interventions, and policies are decided upon. In their qualitative study examining the ethics of global research collaborations, Parker and Kingori (2016) found that addressing ethical concerns in global research collaborations is not only morally desirable, but is required for collaborative global health research to be both successful and sustainable. The successful functioning of global health research networks and hence the successful production of scientific knowledge was seen by scientists in the global south to include an interweaving of scientific, practical, and moral practices. These include the building and maintaining of trust, paying careful attention to fairness in the recognition of efforts, ensuring that scientists in low-income settings are able to meet their obligations to local communities, and the promotion of mutual respect (Parker and Kingori 2016).

## Advancing Global Health Equity in the Context of COVID-19

Addressing the COVID-19 pandemic will require global efforts in surveillance and public health, bringing together scientists, practitioners, policymakers, governments, and many other global health actors. A substantial bioethics literature exists around global health equity and in particular on the ethical distribution of vaccines and the conceptualization and requirements of global solidarity. In this paper we have argued that global health equity in the context of the COVID-19 response can be further advanced by acknowledging the role that LMICs play in global science, as well as through attention to the ethical aspects of collaborative working and the processes by which scientific knowledge is produced and policies decided (Parker and Kingori 2016).
